# Peer Review of “Medical Brain Drain from Southeastern Europe: Using Digital Demography to Forecast Health Worker Emigration”

**DOI:** 10.2196/34078

**Published:** 2021-11-30

**Authors:** Niamh Humphries

**Affiliations:** 1 Royal College of Physicians of Ireland Dublin Ireland

**Keywords:** digital demography, Google Trends, the emigration of doctors and nurses, medical brain drain, Croatia, demography, brain drain, emigration, doctors, nurses, health care workers, health professionals, health systems, jobs, Germany, personnel, migration, workforce, medical professionals

*This is a peer-review report submitted for the paper “Medical Brain Drain From Southeastern Europe: Using Digital Demography to Forecast Health Worker Emigration.”*

## Round 1 Review

### General Comments

Thank you for the opportunity to review this paper [1]. I think it is potentially a very interesting paper, but it needs more work to bring it to publishable standards.

### Specific Comments

1. Data
  - Have digital traces been used as an indicator of migration before? If so, please cite.
  - I have concerns that some of the Google terms used indicate wider emigration rather than health worker migration.
  - If you want to refer to the wider emigration from Croatia to Germany/Austria (either via digital traces or via secondary data), you need to be clear that this is not health care worker (HW) migration you're referring to. I've compared HW migration with migration more generally in a recent paper [2].
  - As an indication of health migration or HW migration intent, you should only focus on the doctor/nurse–specific information.
  - It would greatly strengthen your paper if you had stronger secondary/official data on the migration of nurses/doctors from Croatia/Bosnia and Herzegovina (B&H)/Serbia to Germany/Austria.
  - Can you get data on the number of Croatian/Bosnian/Serbian–trained nurses/doctors who have joined a nursing/medical register in Germany/Austria?
  - Can you get data on the number of Bosnian/Serbian citizens who have obtained visas to work in Germany/Austria?
  - This data would greatly strengthen your argument that digital traces can provide an early indicator of HW migration.
2. Paper structure
  - The Introduction jumps around between source and destination countries. I would suggest that the paper discusses source countries and destination countries separately.
  - Also, I think it's important to separate out push from pull factors and to be specific about the impact on each of the source countries; for instance, as an EU member state, Croatian doctors and nurses can freely migrate and work in Germany/Austria, but doctors/nurses from Serbia or B&H would need visas to work there, right?
  - The pandemic as a push factor is really interesting and an important issue to raise.
  - Why use the term *Western Balkan* if it does not include Croatia? Better to use the countries that you’re talking about (ie, Croatia, Bosnia and Herzegovinia, and Serbia).
3. Engaging with the wider literature
  - I think that the World Health Organization (WHO) Global Code on the International Recruitment of Health Personnel (2010), which is mentioned on page 11, should be more central to the paper, especially in relation to the concept of sustainability and the need for high-income countries to train and retain sufficient HWs to meet needs (article 5.4).
  - Perhaps the paper also needs to mention the WHO 2006 list or the WHO 2020 safeguard list, which lists countries with critical health care shortages. Your paper makes an interesting contribution in highlighting that these issues are also relevant in European countries (see [3]).
  - In relation to Europe, the paper should connect back to the European Observatory books on HW migration from 2014/2015, which highlighted health worker migration from new EU member states to older EU member states [4].
  - In the Introduction, the paper should also connect with the wider literature on brain drain/health worker migration.

## Round 2 Review

### General Comments

Thank you for the revised manuscript; it is a much stronger paper, and the potential of digital demography in forecasting HW migration is now much clearer.

### Specific Comments

1. Perhaps the title should read: Medical Brain Drain From Southeastern Europe: Using Digital Demography to Forecast Health Worker Emigration.
2. You need to be consistent in the terms used throughout the paper (title/abstract/main text). At present the following terms are used to refer to the same places:
  - Western Balkans and Croatia
  - Southeastern Europe
  - B&H
  - I’d suggest using one term that includes Croatia and/or refer to the individual countries and use it throughout the paper.
  - B&H is misspelled once in the Abstract.
  - In displaying numbers/percentages use either decimals (60.09% or 60,09%) or commas in figures, not both.
  - Throughout the paper, perhaps use *a shortage of* in place of *a lack of*.
3. Measuring health worker mobility is a challenge for most countries (not just Croatia, Bosnia, and Serbia), which is why registration and/or visa data from the destination country is often used as a measure of health worker emigration.
4. On page 4, you say that 65,288 nurses emigrated, and I think you’re saying that there are more Croatian/Bosnian nurses in Germany than in Croatia and Bosnia; are you? Perhaps tighten up this sentence as it’s a strong statement.
5. On page 7, the paragraph beginning *in Austria* is unclear. Rewrite these sentences.
6. In Table 3, perhaps mark the stock data versus the flow data (ie, differentiate between the overall number of nurses in Germany [stock] vs the number of nurses entering Germany [flow]).
7. On page 11, the section beginning *what could the European Union do to address the problem*, I think this should be moved out of the Introduction and into the Discussion.
8. I think you could bring one or two issues out in the Discussion that you’ve mentioned in the text already but could make more of:
  - The issue of the European Union drawing health workers from EU countries (Croatia) and nearby countries (Bosnia, Serbia) is an important issue to raise in the Discussion, as it is a clash between free movement (EU free movement) and the right to health care/need to ensure a health workforce in all regions (as per the WHO Global Code and/or the UN Sustainable Development Goals).
  - I think that this method is a really interesting way of generating timely data on health worker migration. During the pandemic, the *normal* ways of data collection are simply too slow (particularly when EU countries are fast tracking health workers into the European Union). Your method is a really good way of generating timely insights into *intent to migrate* among health workers. As you mention in your paper, this should be useful for policy makers (but obviously only if they respond/react to the data). And then the next question for policy makers is how can they retain health workers during a pandemic? Increased salaries? Improved working conditions? Which links nicely to the section on what the European Union can do.

## Abbreviations

B&H: Bosnia and Herzegovina
HW: health care worker
WHO: World Health Organization

## References

[1] Jurić T (2021). Medical brain drain from southeastern Europe: using digital demography to forecast health worker emigration. JMIRx Med.

[2] Humphries N, Connell J, Negin J, Buchan J (2019). Tracking the leavers: towards a better understanding of doctor migration from Ireland to Australia 2008–2018. Hum Resour Health.

[3] Health workforce support and safeguards list, 2020. World Health Organization.

[4] Health professional mobility and health systems. World Health Organization.

